# Automatic Image-Based Plant Disease Severity Estimation Using Deep Learning

**DOI:** 10.1155/2017/2917536

**Published:** 2017-07-05

**Authors:** Guan Wang, Yu Sun, Jianxin Wang

**Affiliations:** School of Information Science and Technology, Beijing Forestry University, Beijing 100083, China

## Abstract

Automatic and accurate estimation of disease severity is essential for food security, disease management, and yield loss prediction. Deep learning, the latest breakthrough in computer vision, is promising for fine-grained disease severity classification, as the method avoids the labor-intensive feature engineering and threshold-based segmentation. Using the apple black rot images in the PlantVillage dataset, which are further annotated by botanists with four severity stages as ground truth, a series of deep convolutional neural networks are trained to diagnose the severity of the disease. The performances of shallow networks trained from scratch and deep models fine-tuned by transfer learning are evaluated systemically in this paper. The best model is the deep VGG16 model trained with transfer learning, which yields an overall accuracy of 90.4% on the hold-out test set. The proposed deep learning model may have great potential in disease control for modern agriculture.

## 1. Introduction

The plant diseases are a major thread to losses of modern agricultural production. Plant disease severity is an important parameter to measure disease level and thus can be used to predict yield and recommend treatment. The rapid, accurate diagnosis of disease severity will help to reduce yield losses [1]. Traditionally, plant disease severity is scored with visual inspection of plant tissue by trained experts. The expensive cost and low efficiency of human disease assessment hinder the rapid development of modern agriculture [2]. With the population of digital cameras and the advances in computer vision, the automated disease diagnosis models are highly demanded by precision agriculture, high-throughput plant phenotype, smart green house, and so forth.

Inspired by the deep learning breakthrough in image-based plant disease recognition, this work proposes deep learning models for image-based automatic diagnosis of plant disease severity. We further annotate the apple healthy and black rot images in the public PlantVillage dataset [3] with severity labels. To explore the best network architecture and training mechanism, we train shallow networks of different depth from scratch and fine-tune the pretrained state-of-the-art deep networks. The models' capabilities of correctly predicting the disease severity stage are compared. The best model achieves an accuracy of 90.4% on the hold-out test set. Our results are a first step towards the automatic plant disease severity diagnosis.

An overview of the rest of the paper is as follows: Section 2 reviews the literature in this area, Section 3 presents the deep learning proposal, Section 4 describes the methodology, Section 5 presents achieved results and related discussions, and, finally, Section 6 holds our conclusions.

## 2. Related Work

Various studies have found that image-based assessment approaches produce more accurate and reproducible results than those obtained by human visual assessments. Stewart and McDonald [4] used an automated image analysis method to analyze disease symptoms of infected wheat leaves caused by* Zymoseptoria tritici*. This method enabled the quantification of pycnidia size and density, along with other traits and their correlation, which provided greater accuracy and precision compared with human visual estimates of virulence. Barbedo [5] designed an image segmentation method to measure disease severity in white/black background, which eliminated the possibility of human error and reduced time taken to measure disease severity. Atoum et al. [6] proposed a novel computer vision system, Cercospora Leaf Spot (CLS) Rater, to accurately rate plant images in the real field to the United States Department of Agriculture (USDA) scale. The CLS Rater achieved a much higher consistency than the rating standard deviation of human experts. Many of these image-based assessment approaches for plant diseases share the same basic procedure [5–13]. Firstly, preprocessing techniques are employed to remove the background and segment the lesion tissue of infected plants. After that, discriminative features are extracted for further analysis. At last, supervised classification algorithms or unsupervised cluster algorithms are used to classify features according to the specific task. Along with advances in computer science, many interactive tools are developed. The Assess [14] is the most commonly used and also the discipline-standard program to estimate disease severity. The Leaf Doctor app [15], developed as an interactive smartphone application, can be used on color images to distinguish lesion areas from healthy tissues and calculate percentage of disease severity. The application achieved even higher accuracy than the Assess.

But these aforementioned plant disease severity estimation approaches are semiautomatic because they depend heavily on series of image-processing technologies, such as the threshold-based segmentation of the lesion area and hand-engineered features extraction. There is usually great variance in color both between lesions of different diseases and between lesions from the same disease at different stages. Therefore, it is very difficult to determine the appropriate segmentation threshold for plant disease images without human assistance. What is more, the time consuming hand-crafted feature extraction should be performed again for new style images. To the best of our knowledge, completely automatic image-based plant disease severity estimation method using computer vision has not yet been reported.

The deep learning approach leads a revolution in speech recognition [16], visual object recognition [17], object detection [18, 19], and many other domains such as drug discovery [20], genomics [21], and building reorganization [22]. Deep learning is very promising for automatically grading plant disease severity. Recently, there have been some works using deep learning method for plant species identification and plant disease identification. The recent years of the well-known annual plant species identification campaigns PlantCLEF [23] were performance-wise dominated by deep learning methods. Choi [24] won the PlantCLEF 2015 by using the deep learning model GoogleNet [25] to classify 1000 species. Mehdipour Ghazi et al. [26] combined the outputs of GoogleNet and VGGNet [27] and surpassed the overall validation accuracy of [24]. Hang et al. [28] won the PlantCLEF 2016 by the enhanced VGGNet model. For plant disease identification, Sladojevic et al. [29] created a dataset with more than 3,000 images collected from the Internet and trained a deep convolutional network to recognize 13 different types of plant diseases out of healthy leaves. Mohanty et al. [30] used a public dataset PlantVillage [3] consisting of 54,306 images of diseased and healthy plant leaves collected under controlled conditions and trained a deep convolutional neural network to identify 14 crop species and 26 diseases. In comparison with classification among different diseases, the fine-grained disease severity classification is much more challenging, as there exist large intraclass similarity and small interclass variance [31]. Deep learning avoids the labor-intensive feature engineering and threshold-based segmentation [32], which is promising for fine-grained disease severity classification.

## 3. Deep Learning Proposal

### 3.1. Deep Convolutional Neural Network

To explore the best convolutional neural network architecture for the fine-grained disease severity classification problem with few training data, we compare two architectures, namely, building a shallow network from scratch and transfer learning by fine-tuning the top layers of a pretrained deep network.

The shallow networks consist of only few convolutional layers with few filters per layer, followed by two fully connected layers, and end with a softmax normalization. We train shallow networks of 2, 4, 6, 8, and 10 convolutional layers. Each convolutional layer has 32 filters of size 3 × 3, a Rectified Linear Units (ReLU) activation, and all layers are followed by a 2 × 2 max-pooling layer, except for the last convolutional layer, which has 64 filters. The first fully connected layer has 64 units with a ReLU activation and is followed by a dropout layer with a dropout ratio of 50%. The last fully connected layer has 4 outputs, corresponding with the 4 classes, which feed into the softmax layer to calculate the probability output.

### 3.2. Transfer Learning

**Figure 1:**
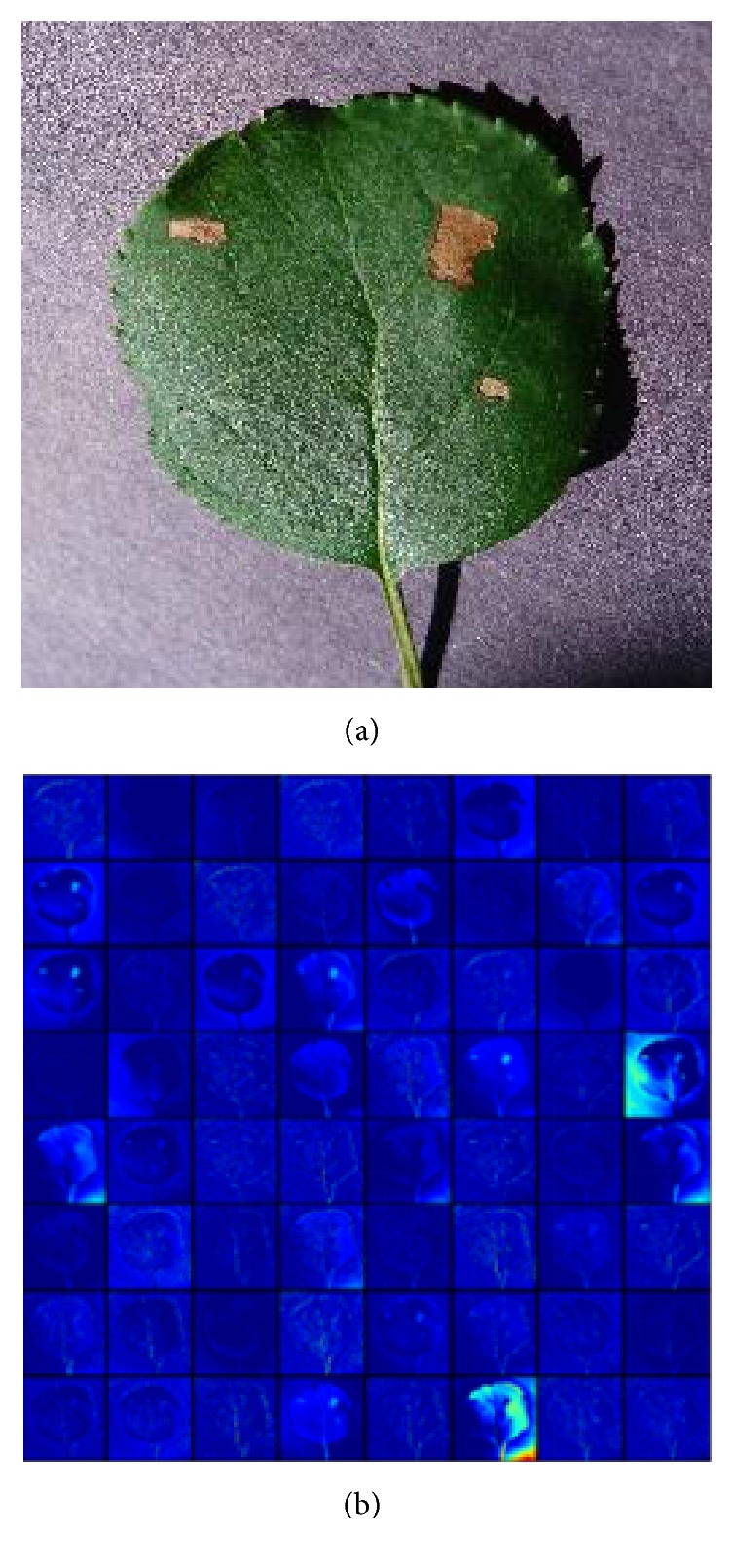
Visualization of activations for an input image in the first convolutional layer of the pretrained VGG16 model: (a) original image; (b) the first convolutional layer output.

It is notable that the amount of images we can learn from is quite limited. Transfer learning is a useful approach to build powerful classification network using few data, by fine-tuning the parameters of a network pretrained on a large dataset, such as ImageNet [26]. Although the disease severity classification is targeted for finer grained image category classification problem compared to the ImageNet, the lower layers only encode simple features, which can be generalized to most computer vision tasks. For example, the first layer only represents direction and color, and the visualization of activations in the first layer of VGG16 model is shown in Figure 1. Though not trained on the plant disease dataset, the model can be activated against the diseased spots, the leaf, and the background.

For transfer learning, we compare the VGGNet [27], Inception-v3 [33], and ResNet50 [17] architectures. VGGNet and the original Inception architecture GoogleNet yielded similar high performance in the 2014 ImageNet Large Scale Visual Recognition Challenge (ILSVRC), and ResNet won the first place of the challenge in 2016. The VGGNet involves 16 (VGG16) and 19 (VGG19) weight layers and shows a significant improvement on prior configurations by using an architecture with very small convolution filters. The original Inception architecture GoogleNet combines the network-in-network approach and the strategy of using a series of filters of different sizes to handle multiple scales. The Inception-v3 is an improved Inception architecture which can be scaled up with high computational efficiency and low parameter count. ResNet is built up by stacking residual building blocks. Each building block is composed of several convolutional layers with a skip connection. It lets each stacked layer fit a residual mapping, while skip connections carry out identity mapping. It is easier to optimize the residual mapping than to optimize the original mapping. The architecture solves the degeneration problem: as stacking more layers, the accuracy gets saturated and then degrades rapidly. ResNet50 is the 50-layer version of the network.

## 4. Material and Experiment

### 4.1. Data Material

**Figure 2:**
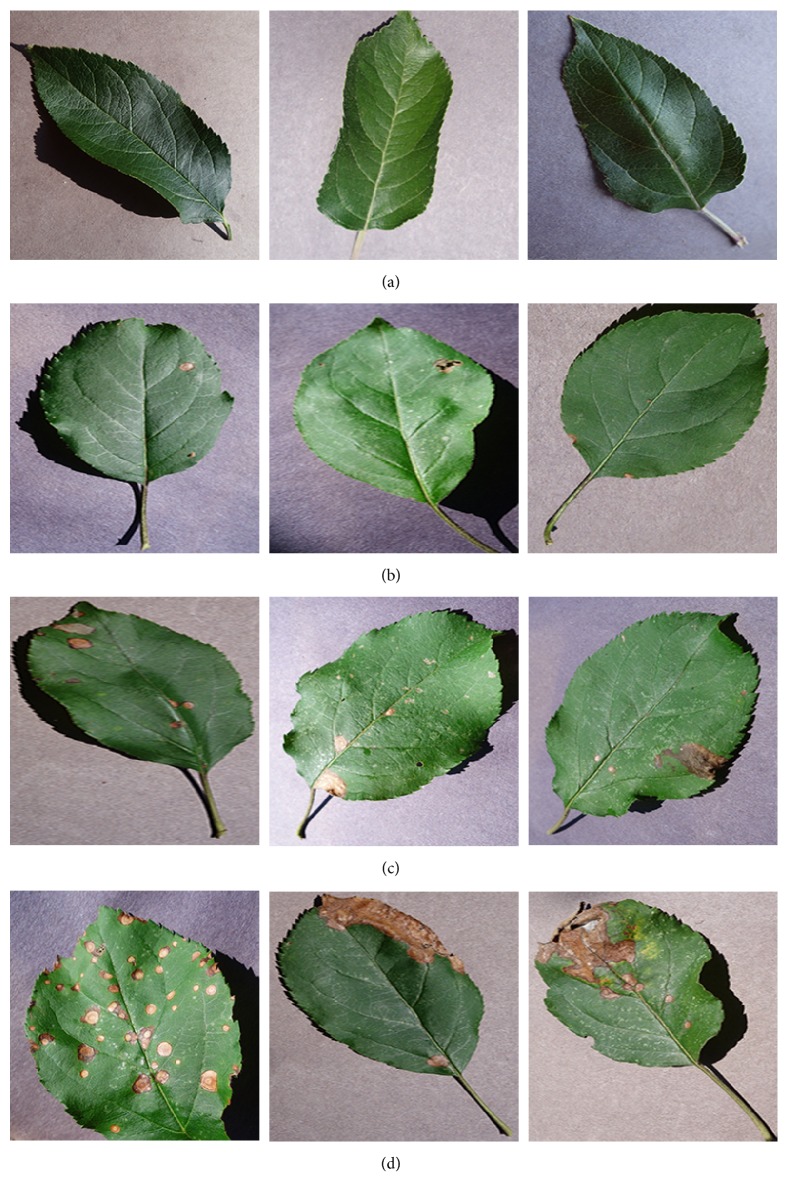
Sample leaf images of the four stages of apple black rot: (a) healthy stage, (b) early stage, (c) middle stage, and (d) end stage.

The PlantVillage is an open access database of more than 50,000 images of healthy and diseased crops, which have a spread of 38 class labels. We select the images of healthy apple leaves and images of apple leaf black rot caused by the fungus* Botryosphaeria obtusa*. Each image is assessed into one class by botanists: healthy stage, early stage, middle stage, or end stage. The healthy-stage leaves are free of spots. The early-stage leaves have small circular spots with diameters less than 5 mm. The middle-stage leaves have more than 3 spots with at least one frog-eye spot enlarging to irregular or lobed shape. The end-stage leaves are so heavily infected that will drop from the tree. Each image is examined by agricultural experts and labeled with appropriate disease severity. 179 images which are inconsistent among experts are abandoned.  Figure 2 shows some examples of every stage. Finally, we get 1644 images of healthy leaves, 137 early-stage, 180 middle-stage, and 125 end-stage disease images.

As healthy leaves are much more than the diseased leaves, there is much difference in the number of samples per class. The number of samples per class should be balanced to reduce the bias the network may have towards the healthy-stage class with more samples. Our strategy of balancing is as follows: for early stage, middle stage, and end stage, about 80% of the images are used as the training set and the left 20% are the hold-out test set. For healthy-stage leaves, the images are divided into 12 clusters, with 110 images in each cluster on average for training. 27 images are left for testing. The final accuracy is estimated by averaging over 12 runs on the clusters. As the PlantVillage dataset has multiple images of the same leaf taken from different orientations, all the images of the same leaf should be either in the training set or in the test set.  Table 1 shows the number of images used as training and test sets for each class.

### 4.2. Image Preprocessing

The samples in the PlantVillage dataset are arbitrarily sized RGB images. Thanks to the powerful end-to-end learning, deep learning models only need 4 basic image preprocessing steps. Images are processed according to the following stages: firstly, we resize all the images to 256 × 256 pixels for shallow networks, 224 × 224 for VGG16, VGG19, and ResNet50, and 299 × 299 for Inception-V3. We perform both the model optimization and prediction on these rescaled images. Secondly, all pixel values are divided by 255 to be compatible with the network's initial values. Thirdly, sample-wise normalization is performed. Normalization can significantly improve the efficiency of end-to-end training. The normalization is performed as follows: for each input *x*, we calculate the mean value *m*_*x*_ and standard deviation *s*_*x*_ and then transform the input to *x*′ = (*x* − *m*_*x*_)/*s*_*x*_, so that the individual features more or less look like standard normally distributed data with zero mean and unit variance. Finally, several random augmentations including random rotation, shearing, zooming, and flipping are applied to the training images. The augmentation prevents overfitting and makes the model generalize better.

### 4.3. Neural Network Training Algorithm

The basic architecture in the convolutional neural network begins with several convolutional layers and pooling layers, followed by fully connected layers. For an input *x* of the *i*th convolutional layer, it computes

(1) $$\begin{array}{c} x_{ic}=ReLU\left(\;W_{i}*x\;\right), \end{array}$$

where *∗* represents the convolution operation and *W*_*i*_ represents the convolution kernels of the layer. *W*_*i*_ = [*W*_*i*_^1^, *W*_*i*_^2^,…, *W*_*i*_^*K*^], and *K* is the number of convolution kernels of the layer. Each kernel *W*_*i*_^*K*^ is an *M* × *M* × *N* weight matrix with *M* being the window size and *N* being the number of input channels.

ReLU represents the rectified linear function ReLU(*x*) = max⁡(0, *x*), which is used as the activation function in our models, as deep convolutional neural networks with ReLUs train several times faster than their equivalents with saturating nonlinearities.

A max-pooling layer computes the maximum value over nonoverlapping rectangular regions of the outputs of each convolution kernel. The pooling operation enables position invariance over larger local regions and reduces the output size.

Fully connected layers are added on top of the final convolutional layer. Each fully connected layer computes ReLU(*W*_fc_*X*), where *X* is the input and *W*_fc_ is the weight matrix for the fully connected layer.

The loss function measures the discrepancy between the predicted result and the label of the input, which is defined as the sum of cross entropy:

(2) EW=−1n∑xi=1n ∑k=1Kyiklog⁡Pxi=k+1−yiklog⁡1−Pxi=k,

where *W* indicates the weight matrixes of convolutional and fully connected layers,* n* indicates the number of training samples,* i* is the index of training samples, and *k* is the index of classes. *y*_*ik*_ = 1 if the *i*th sample belongs to the* k*th class; else *y*_*ik*_ = 0. *P*(*x*_*i*_ = *k*) is the probability of input *x*_*i*_ belonging to the* k*th class that the model predicts, which is a function of parameters *W*. So the loss function takes *W* as its parameters.

Network training aims to find the value of *W* that minimizes the loss function *E*. We use gradient descent algorithm where *W* is iteratively updated as

(3) $$\begin{array}{c} W_{k}=W_{k-1}-\alpha \frac{\partial E\left(W\right)}{\partial W}, \end{array}$$

where *α* is the learning rate, which is a very important parameter that determines the step size of the learning. The value of learning rate should be carefully evaluated.

We use early stopping as the training stop strategy to stop training when the network begins to overfit the data. The performance of the network is evaluated at the end of each epoch using the test set. If the loss value of the test set stops improving, the network will stop training.

To prevent overfitting, the transfer learning is conducted as follows: fully connected layers are replaced with a new one and only fine-tune the top convolutional block for VGG16 and VGG19, the top two inception blocks for Inception-v3, and the top residual block for ResNet50, along with the new fully connected layers. To avoid triggering large gradient updates to destroy the pretrained weights, the new fully connected network should be initialized with proper values rather than with random values. So firstly we freeze all layers except the new fully connected network. The new fully connected network is trained on the output features of the final convolutional layer. The weights learned from training are initial values for fine-tuning. After that, the top convolutional block for VGG16 and VGG19, the top two inception blocks for Inception-v3, and the top residual block for ResNet50 are unfreezed and then trained along with the new fully connected network with a small learning rate.

The parameters for training shallow networks and fine-tuning pretrained models are presented in Table 2. Besides, a learning rate schedule is employed. The initial learning rate is dropped by a factor of 10 every 50 epochs for training shallow networks with less than 6 convolutional layers and fine-tuning deep networks. And it dropped by 10 every 100 epochs for shallow networks with 6 or more convolutional layers. Because the network goes deeper, it needs more training steps to converge.

### 4.4. Implementation

The experiment is performed on an Ubuntu workstation equipped with one Intel Core i5 6500 CPU (16 GB RAM), accelerated by one GeForce GTX TITAN X GPU (12 GB memory). The model implementation is powered by the Keras deep learning framework with the Theano backend.

## 5. Result and Discussion

**Figure 3:**
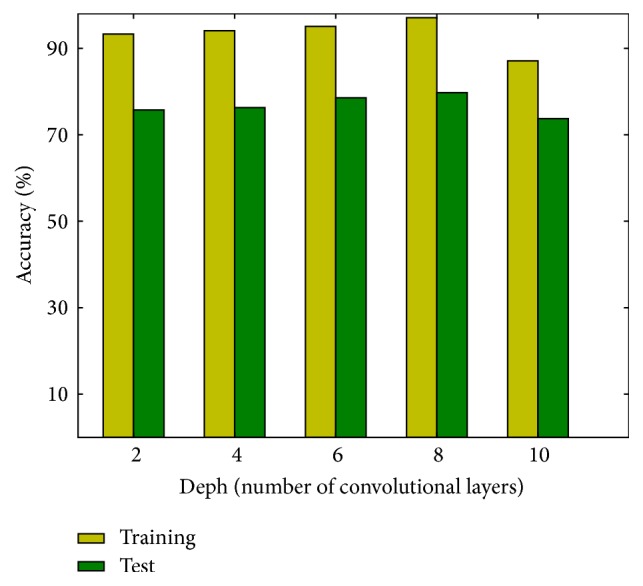
Accuracies of shallow networks.

Figure 3 shows the training and testing accuracies of shallow networks trained from scratch. Each bar represents the average result of 12 runs. Both training and test accuracies improve slightly with the depth of the model at first. The best performance, that is, a test accuracy of 79.3%, is achieved by the network with 8 convolutional layers. But the accuracies fall when the network's depth exceeds 8, as there are insufficient training data for models with too many parameters. To circumvent this problem, transfer learning is applied to the state-of-the-art deep models.

**Figure 4:**
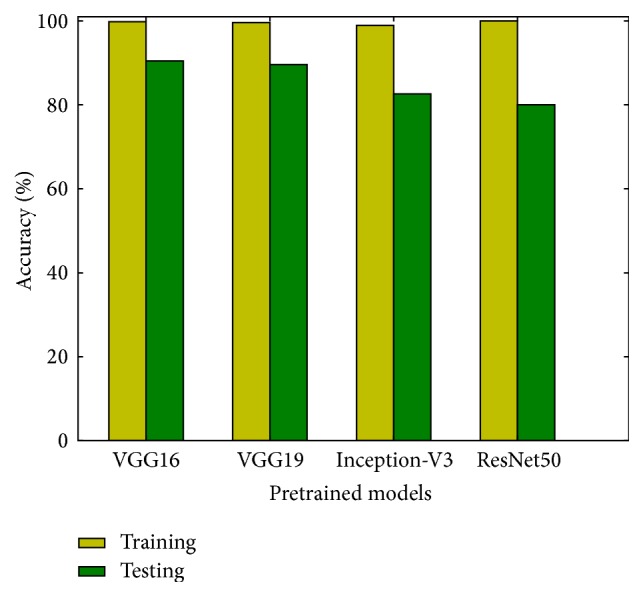
Accuracies of the state-of-the-art extreme deep models trained with transfer learning.

The results of fine-tuning the ImageNet pretrained models are reported in Figure 4. Each bar represents the average result of 12 runs. The overall accuracy on the test set we obtained varies from 80.0% to 90.4%. The performance of fine-tuned models is superior to that of models trained from scratch. The best result is achieved by the VGG16 model, with an accuracy of 90.4%. The results indicate that transfer learning alleviates the problem of insufficient training data.

For comparison, an ANN model is trained by SGD optimizer end-to-end on the training set. A test accuracy of 31% is achieved, which is basically random guessing. Without the convolutional feature extractor, the ANN cannot extract local correlations and learn discriminative features from the images.

The confusion matrix of the VGG16 model on the hold-out test set is shown in Table 3. The fraction of accurately predicted images for each of the four stages is displayed in detail. All of the healthy-stage leaves are correctly classified. The accuracies of early stage and end stage are 93.1% and 87.0%, respectively. Middle stage is prone to be misclassified, with an accuracy of 83.3%. However, the misclassified stages are only confused with their adjacent stages. For example, the early stage is only confused with the middle stage, and none of early-stage is classified as end stage.

From the results displayed in Figure 4, it is notable that the training accuracies of deep networks are close to 100% and trigger the early stopping. Since deep learning is data-driven, training on more data will further increase the test accuracy. It is also important to note that the best performance is achieved by the VGGNet. The result is consistent with that of [26, 28], where the VGGNet showed better performance in the PlantCLEF plant identification task. Though ResNet achieved state-of-the-art result on the ImageNet dataset, it performs poorer than VGGNet on fine-grained classification tasks. The SGD optimizer might put the residual mapping in building blocks of ResNet to zero too early, which leads to a local optimization and results in the poor generalization in fine-grained classification.

## 6. Conclusion

This work proposes a deep learning approach to automatically discover the discriminative features for fine-grained classification, which enables the end-to-end pipeline for diagnosing plant disease severity. Based on few training samples, we trained small convolutional neural networks of different depth from scratch and fine-tuned four state-of-the-art deep models: VGG16, VGG19, Inception-v3, and ResNet50. Comparison of these networks reveals that fine-tuning on pretrained deep models can significantly improve the performance on few data. The fine-tuned VGG16 model performs best, achieving an accuracy of 90.4% on the test set, demonstrating that deep learning is the new promising technology for fully automatic plant disease severity classification.

In future work, more data at different stages of different diseases will be collected with versatile sensors, like infrared camera and multispectral camera. The deep learning model can be associated with treatment recommendation, yield prediction, and so on.

## Figures and Tables

**Table 1:** The number of samples in training and test sets.

Class	Number of images for training	Number of images for testing
Healthy stage	110 × 12	27 × 12
Early stage	108	29
Middle stage	144	36
End stage	102	23

**Table 2:** The hyperparameters of training.

Parameters	Learning from scratch	Transfer learning	
		Training fully connected layers	Fine-tuning
Training algorithm	SGD	RMSP	SGD
Learning rate	0.01	0.01	0.0001
Batch size	32		
Early stopping	10 epochs		

**Table 3:** Confusion matrix for the prediction of VGG16 model trained with transfer learning.

	Predicted				
Ground truth		Healthy stage	Early stage	Middle stage	End stage
	Healthy stage	27	0	0	0
	Early stage	0	27	2	0
	Middle stage	0	5	30	1
	End stage	0	0	3	20
